# Adding Handles to Optimize Manual Box Handling

**DOI:** 10.1155/2018/7315217

**Published:** 2018-11-18

**Authors:** Helen Cristina Nogueira, Leticia Bergamin Januario, Roberta de Fátima Carreira Moreira, Ana Beatriz Oliveira

**Affiliations:** Laboratory of Clinical and Occupational Kinesiology (LACO), Department of Physical Therapy, Federal University of São Carlos, Brazil

## Abstract

The risk factors for developing musculoskeletal disorders in material handling tasks are well known. Among strategies for controlling risks, modifying boxes by adding handles is suggested. However, there are no clear recommendations regarding box modification as an approach to improve musculoskeletal health. In this study, we investigated the main literature databases to identify effects of box modification on reducing physical load. Electronic and manual searches were performed to identify studies that evaluated effects of boxes handles on physical exposure during handling tasks. The included studies were very heterogeneous (methods of assessment, types of handles used, and methodological quality), jeopardizing synthesis of evidence. Despite the mentioned limitations, we could suggest some features that could improve manual handling in practical settings, like the use of cylindrical handles forms with intermediate diameters (between 31 and 51 mm) and 30° inclination. Those characteristics demonstrated positive results on physical exposure. Regular cut-outs were indicated as a beneficial approach when boxes are handled in high surfaces. When handling occurs in medium heights or in the floor level, handles positioned on the top of the box might bring better results. Efforts to standardize methods are important to support both objective and subjective assessment of box handle design, as well to improve the internal validity of studies.

## 1. Introduction

Several studies have demonstrated associations between manual material handling (MMH) tasks and work related musculoskeletal disorders (WMSDs) [1–6]. Manual material handling is still an extremely common and essential activity in industrialized and developing countries. Therefore, research that supports the control of risk factors for WMSDs inherent to MMH has great social value.

The physical load imposed during handling tasks is a consequence of mechanical demands such as arm elevation above the shoulder height, back or spinal flexion combined with trunk rotation movements, heavy loads, and high task repeatability [5, 7]. More careful transportation segments (such as fresh produce handling, handling delicate materials such as electronic components, and many others), where the materials being handled are both heavy and delicate, may disproportionately contribute to the problem of WMSDs [8–12].

As an approach to controlling WMSDs, some studies suggested adding handles to boxes [13, 14]. Handles are proposed to improve the coupling of the hands with the box and control large wrist deviation, demonstrating biomechanical and physiological advantages compared to boxes without any modification [13, 15–17]. Studies have shown that there is a greater hand force exertion and higher stability on containers with handles [5, 18, 19]. Moreover, the insertion of handles can be considered an economically feasible intervention when compared with the high cost of task mechanization.

Drury [20] reviewed all available studies involving handles and concluded that the workstation adjustment and the subjects' anthropometrics were correlated to provide good working conditions. Moreover, there are no clear recommendations regarding how to perform handle adjustments on the boxes, such as the impact of this approach on controlling musculoskeletal disorders [21]. Considering the high prevalence of WMSDs in industrialized countries as well as the fact that handling tasks are an important work activity, this review summarizes the results from available studies involving box modification by adding handles and provides information to optimize box handle adoption.

## 2. Methodology

### 2.1. Search Strategy

A list of articles dating as far back as 1980 was compiled using a series of keywords with six databases (Embase, Pubmed/Medline, Web of Science, Bireme, Lilacs, and CINAHL SPORTDiscus). The keywords were organized as a string ((box OR environment design) AND (“manual handling” OR “manual material handling” OR “weight lifting” OR handles OR “hand strength”) AND workers). No previous reviews containing this topic were identified in the literature. The literature search was performed including titles published until December 2017. The electronic search resulted in a total of 1170 references published in English, of which 570 were repeated, offering 600 titles for analysis. Of those, 80 articles were selected by consensus to have their summaries read. Thirteen studies were considered relevant enough for complete reading, of which six were excluded for failing to evaluate the effect of modification of boxes by adding handles. Through manual search of the reference lists of the thirteen accepted articles, six additional articles were included, three of which had two studies with different methodologies in the same publication [13, 22, 23], for a total of 17 studies included in this review.

### 2.2. Selection Process

This systematic review considered papers published in English. Articles published in conference proceedings, books, or book chapters, as well as research reports, were not considered. The intervention had to be aimed at the prevention of musculoskeletal problems through modifying boxes by adding handles. Changes resulting from the intervention had to involve changes in work tasks as opposed to, for example, changes in organizational structure. We considered papers that evaluated both workers experienced with handling activities and nonexperienced subjects, regardless of gender. The present study included papers that evaluated the design of the handles and those focused on assessing the position of the handles in the boxes.

### 2.3. Analysis Strategy for Selected Publications

To extract the major information from the selected articles, we defined three factors to describe how to reproduce box modification as an intervention during handling task: (1) handle form description; (2) handle location in boxes; (3) assessment measures.

Additional information, such as the subject's occupational activity, manual dominance, body part assessed, work environment (simulated versus real), general layout of the experiment equipment, and weight of the boxes, was also collected.

At first, two independent reviewers selected the studies based on titles, excluding those clearly unrelated to the subject of the review. Next, all selected titles had their abstracts were analyzed to identify those that could be included considering the previous criteria. The relevant abstracts were evaluated independently by the same two reviewers. Disagreements during the selection process and assessment were resolved by consensus. When consensus has not been obtained by two reviewers a third reviewer was consulted to make the final judgment. This assessment phase of the studies was performed using the StArt software (State of the Art through Systematic Review) v.1.06.2. The reference lists of all included studies were also checked, through the snowball method, in order to identify possible studies not retrieved by the electronic search.

## 3. Results

Results are mainly presented through tables, which include data in raw form. Subjects' anthropometric aspects, samples size, and occupational role are presented in Table 1. Occupational role is balanced between students and workers. The majority of participants were males, middle aged, with no manual dominance reported, and no self-reported musculoskeletal symptoms.

Box size, handle designs, measurement methods, and areas of the body measured are presented in Table 2. In general, the selected studies had handle descriptions regarding size, material, and position in the boxes. The tools of assessment used were diverse across the studies.

## 4. Discussion

This study included 17 cross-sectional studies that evaluated the implementation of handles in boxes from 1980 until 2016. We identified a wide variety of assessment tools used in the included studies. Specially regarding the evaluation of objective measurements, due to the advances in technological devices over time.

### 4.1. Handles Design

Previous studies intended to investigate which handle features, such as diameter and shape, had better results during handling boxes [13, 22, 24]. The selected studies did not identify differences on perceived exertion regarding the handle shape, except in Drury et al. [13], which identified positive effects of cylindrical forms. Drury et al. [13] also observed that intermediate diameters, between 31 and 51 mm, presented better rates of perceived exertion and discomfort, regardless of a straight or curved shape.

The preferences for a specific wrist posture were discussed and depended on the height of box deposition according to Drury et al. [20]. The most used handling height was considered important to determine handle angle considering the risks to increase the perception of effort when the handles were not positioned for biomechanical advantage on the box. On the other hand, Shih and Wang [24] recommended inclinations between -10° and 10° to allow neutral wrist posture (0° of deviation) for both genders evaluated. The most recently published paper from Silva et al. [25] observed that 30° inclination in handles enabled safer scores on all variables tested at different heights of handling. With better methodological quality, Silva et al. [25] used different measurement methods that were complementary in evaluating the upper limbs during handling with different grip designs.

### 4.2. Positioning of the Handles on the Box

Findings regarding the best handle positions are heterogeneous and conflicting. Asymmetric handle placement improved the scores of effort and discomfort, when compared to symmetric placement [22, 23] (Deeb and Drury, 1985). Studies that evaluated exertion showed positive effects of asymmetric handles with distinct recommendations about the location of those handles on the boxes [18, 23]. Deeb et al. [22] evaluated forces imposed on the spine and concluded that symmetric handles resulted in lower load in the lumbar spine when compared to asymmetric ones. On the other hand, the symmetry did not influence subjects' heart rate and worsened scores of perceived exertions. Considering the importance of manual dominance in determining the placement of the hand, this lack of information in several studies can be considered a limitation to safe recommendations.

Some studies suggested that the grip asymmetry during load handling can lead to an overload on specific musculoskeletal structures, restrict force generation, and increase the load on the spine [26, 27]. However, Gagnon [28] observed biomechanical advantages for the lumbar spine during asymmetric hand position combined with box tilting when evaluating experienced subjects. This approach of tilting the boxes enables better alignment of the hip, spine, and shoulder, with a smaller range of flexion motion in knee and column joints, which provides more effective and safer acceleration and deceleration movements during box handling [29]. On the other hand, inexperienced subjects do not usually tilt the box and, during handling with asymmetrical grips, their knees and spine flexion motions are greater and their perceived physical load is worse than experienced workers, due to higher energy expenditure during box acceleration and deceleration. Thus, hand asymmetry itself could increase the overload risk of multiple joints due to the restriction of box motions observed in inexperienced subjects. The intrinsic motor variability between subjects in combination with task demands makes it impossible to suggest an ideal physical technique for all working conditions.

Studies that evaluated biomechanical exposure of the lumbar spine with modern methods [5] (Davis and Marras 1998) demonstrated benefits for handles inserted at the upper part of the box in all handling heights. This handle positioning was associated with the reduction of spinal load due to smaller ranges of flexion, corresponding to a reduction of 4.5 kg of load on the lumbar region. On the other hand, more recent studies [11, 14, 25, 30], which evaluated load on upper limbs, suggested that handle modifications should be made according to the handling height. Handles located at the upper part of the box may create higher demands on upper limbs when the load is placed in higher places.

Silva et al. [30] demonstrated advantages using cut-out handles as Drury [20] suggested. In palletizing tasks, handles that are outside of the box may hinder the usefulness of storage boxes. Silva et al. [30] also considered the high usability of cut-out handles despite the reduction of box volume associated with handles design. The latest selected study [11], which evaluated subjects of differing work experience levels, suggested boxes with handles mainly had advantages in musculoskeletal load and perceived exertion for nonexperienced subjects.

Finally, it is also important to consider the boxes weight during MMH and evaluation of musculoskeletal load [31]. Even though, the weight assessment was not an aim in this study, it is important to consider its relevance when evaluating methods to control the physical risks.

### 4.3. Methods of Assessment

Assessment of specific anatomical structures and the use of tools with valid and reliable measures can explain the real conditions of working tasks [32–34]. In general, the lack of description of methodological procedures decreased the external validity of the included studies. Only studies published from 1997 have provided reproducible descriptions.

### 4.4. Limitations

- Heterogeneity of the studies did not allow the search for articles by electronic databases alone. Searches of bibliographic references were widely used for selecting studies. The lack of keywords in earlier studies could explain this problem.
- All the studies included in this review were cross-sectional. This model does not allow to establish a cause-effect relationship, since the measures are taken only once on the time line, making it not possible to consider the effect of box modification over time.
- The methodological quality was assessed according to Ariens et al. [35], being specific for studies with cross-sectional design. Nevertheless, we recognize that this scale contains items that are not related to the objectives of this review.
- The primary studies included in this review were mostly performed in laboratory settings. We do not have a picture of how and how often handles have been used in real occupational settings. From our practice, we can say that cut-outs tend to be more common in cardboard boxes (particularly those used to pack heavy and large products), while plastic boxes used in various occupational contexts (warehouse, supermarkets, industry, etc.) tend to have different handles format. Further research, performed in real occupational settings, will be important to evaluate the feasibility of implementing handles as recommend in this study.

## 5. Conclusion

Considering the high methodological variability of the studies and technological advances in research instrumentation, it is difficult to objectively synthetize evidence to practical ergonomics. Despite this limitation, we could synthetize some practical recommendations that could improve manual handling. The use of cylindrical forms with intermediate diameters (between 31 and 51 mm) and 30° inclination has demonstrated positive results on physical exposure. We suggest that handles could improve working conditions since they could be inserted according to task demands. Regular cut-outs were indicated as a beneficial approach when boxes are handled in high surfaces. When handling occurs in medium heights or in the floor level, handles positioned on the top of the box might bring better results. Handles can be beneficial particularly for inexperienced subjects. Furthermore, improvements in methodological quality including the use of direct measurements to evaluate physical exposure and more detailed and standardized descriptions should be also considered in future research. Further studies shall look at the feasibility of implementing handles as a strategy to decrease musculoskeletal load.

## Figures and Tables

**Table 1:** Participants characteristics identified from the articles included in this study.

**Paper**	**Sample size **	**Occupational activity**	**Gender**	**Age ± SD (years) **	**Weight (Kg)**	**Height (cm)**	**Manual dominance**	**Musculoskeletal symptoms**
Drury et al. 1980-1	7	Workers	Men	21 to 37	not reported	not reported	not reported	no
Drury et al. 1980-2	12	Students	Men	not reported	not reported	not reported	not reported	no
Coury et al. 1982	10	Students	Men	21.3±1.2	78.8±10.6	179.3±5.9	right handed	no
Deeb et al. 1985	30	Workers	15 women	M:23.5±3.2	M:78.7±9.39	M:180±0.64	not reported	no
			15 men	W: 25.9±9.73	W:64.3±10.16	W:166±0.53		
Deeb et al. 1986-1	10	Students	5 women	not reported	M:73.06	M:180	not reported	no
			5 men		W: 57.75	W:163		
Deeb et al. 1986-2	6	Workers	Men	31.3±17.91	89.5±20.14	179±9	not reported	no
Drury et al. 1986 – 1	30	Workers	15 women	M 23.5±3.20	M:78.7±9.39	M180±0.64	not reported	no
			15 men	W: 25.9±9.73	W:64.3±10.16	W166±0.53		
Drury et al. 1986 – 2	30	Workers	15 women	M: 23.5±3.20	M:78.7±9.39	M180±0.64	not reported	no
			15 men	W: 25.9±9.73	W:64.3±10.16	W166±0.53		
Ciriello et al. 1993	6	Workers	Men	40.5 ± 20.3	81.8±14.6	174.4 ± 8.1	not reported	no
Shih et al. 1997	12	Students	6 women	19	not reported	not reported	not reported	no
			6 men					
Davis et al. 1998	10	Workers	Men	19 a 49	80.1±8.4	180.3±2.3	not reported	no
Marras et al. 1999	10	Workers	Men	27.2	89.1±8.4	180.3 ± 7.1	not reported	no
Ando et al. 2000	11	Students	Men	20.5 ± 2.5	60.6±7.3	170.2 ± 4.7	right handed	no
Jung et al. 2010	20	Students	Men	24.78±0.7	66.65±6.73	173.75±4.69	not reported	no
Silva et al. 2012	10	Workers	Men	38.2 ± 8.3	78.3±13.2	170 ± 0.5	right handed	no
Silva et al. 2013	37	Students	Men	23.85±3.97	73.95±10.35	170±30	right handed	no
Nogueira et al. 2016	21	Workers	Men	29.39±6.45	81.36±13.36	170±60	right handed	no

**Table 2:** Used handles dimension, boxes' characteristic, type of the measurements, body regions evaluated, and relevant findings.

**Trial**	**Handles Dimension**	**Boxes' characteristic**	**Measurements **	**Body part**	**Result**
**Handles Design**					
Drury et al, 1980-1	7 types: 5 cylindrical, 1 rope and 1 flat circle	42.5x20.0x39.5	Scale of working time duration	Not reported	The recommendation was cylindrical handles with metal counterpart.
Drury et al, 1980-2	14 types with different diameters	not reported	Dynamometer, Comfort scale (Salvaterra and Chiusano 1978)	Not reported	The optimum diameter related was at 31 to 38mm.
Deeb et al, 1986-1	Wood handles with 2,5cmX10 and diameter of 2.5 cm.	39.5x21.5x42.5	Borg scale (Borg 1962)	All body	The results show that there were no differences in the variables between the forms of handle (straight or curved). Already the smallest thickness of the handle had a negative impact on variables
Deeb et al, 1986-2	Curve handles thick 150mm and forwarded to the radio	40.0x40.0x40.0	Gravity roller conveyor system, cameras, Beckman Dynographic, Borg scale (Borg 1962)	Elbow, wrist and spine	Handle shape was not significant for any of the variables. Handle position symmetrical had a significant effect on both elbow angles, left wrist angle, and disc comprehensive force.

**Handles position**					
Coury et al, 1982	10 types coupled in different positions	40.0x40.0x40.0; 40.0x40.0x50.0; 45.0x40.0x45.0; 50.0x40.0x40.0; 50.0x40.0x50.0	dynamometer, Beckman model, Borg scale (Borg 1962)	Hands	The force production decreasing as the handles was used. The HR measures and the subject's perception of effort were similar with higher values in the symmetric handles in the top of the box. The recommendations varied according to the handle combination 1, 2 and 4
Deeb et al, 1985	9 types coupled in different positions	40.0x40.0x40.0	dynamometer and camera, Borg scale (Borg 1962), Corlett and Bishop's (1976) scale, Body part discomfort severity (BPDS)	All body	Handle positions 3/8 and 6/8 give clearly lower heart rates than the other positions for handling in floor and waist levels and the symmetrical positions (2/2 and 8/8) for heavy containers near floor level. Handle angle should be about 70° for all handles. Although the positions 3/8 and 6/8 are preferred in the subjective scales, symmetric grip has lower power peaks.
Drury et al, 1986 - 1	5 types in different positions	40.0x40.0x40.0	Films and Beckman Model R611 polygraph	Upper limbs	Optimum box handle positions were 6/8, 3/8 for most lifts. Position 2/2 was useful for heavy boxes at floor level.
Drury et al, 1986 - 2	9 types coupled in different positions	40.0x40.0x40.0	Beckman Model R611 polygraph, Borg scale (Borg 1962), Body part discomfort frequency. Body part discomfort severity (Corlett 1976).	Trunk, arms and legs	For the floor level symmetrical handles position 2/2 was better. For handling to the waist and to the shoulder level handle position 3/8 was better, while for the largest distance from the floor to the shoulders levels handle position 3/7 had lower reports of discomfort and HR measurements. The handle position 6 was adequate at 60 degrees of angulation while at hand position 8 indicated 50 degrees of angulation. Benefit of symmetry only for lower handling conditions and asymmetry for the other handling levels.
Ciriello et al, 1993	Wood handles (7.8x4.2 cm) in the top edges of the box	Cardboard: s,m,l. Plastic: 6 dimensions	Dynamometer, bicycle ergometer, Psychophysical scale (Ciriello e Snook 1983)	Trunk	The handles provide greater MAW except at the lowest height and does not interfere in HR and VO2. The size and combination tasks didn't affect the MAW.
Shih et al, 1997	6 diameters: 25.4 mm, 31.8 mm, 38.1 mm, 44.5 mm, 50.8 mm and 57.2 mm. 6 handle angles: radial 20°, 10°, neutral, ulnar 10°, 20° and 30°	25x20x35	Psychophysical scale, AHP 9-point scale (Saaty, 1980.	Hands	The authors recommended handle diameter with 31mm and neutral handle angle.
Davis et al, 1998	8.9x2.5cm in the center side of the box	S: 30.5x40.6x20.3. L: 30.5x49.5x27.5	Lumbar Motion Monitor, EMG, force platform, EGM	Spine	Handle coupling and pallet region were found to significantly influence both
					anterior-posterior shear as well as compression forces. Handles reduced the lateral shear forces in the lower regions. The importance of handles was specially in the lower regions of the pallet deposition. They concluded handles should be adopted.
Marras et al.1999	8.9x2.5	S 20.3x40.6x30.5 L28.0x49.5x30.5	lumbar monitor motion, EMG, force platform	Spine	The inclusion of handles had an effect similar to reducing the box weight by 4.5 kg, whereas box size did not effectively in the spine loading.
Ando et al, 2000	(1) handle type: upper edges of the box. (2) Bottom type: bottom edges on either side. (3) Oblique type: holding the left lower proximal corner and the right upper distal corner	29 cm long, 24.5 cm wide, 23 cm high and 10 kg weight	Dynamometer; Borg scale	Arms, shoulders, back, and thighs	In dynamic lifting Handle type lifting was recommended while in isometric effort oblique type was better.
Jung et al, 2010	11.5x 2.5 x3.8 to 5cm to the box's upper border, in the middle and 5 cm to the box's lower border	30.0x30.0x30.0; 40.0x40.0x40.0; 50.0x50.0x50.0; 60.0x60.0x60.0	Body part discomfort frequency (Corlett 1976).	Neck, shoulder, back, elbow, hand, thigh, knee, ankle	It was recommended that a box provides a handle according to its relevant position, depending on size and manual handling condition.
Silva et al, 2012	9 x 4	27.0x47.0x53.0	Electrogoniometers and films	Upper limbs	The majority of the workers used the cutouts, either symmetrically or asymmetrically. The use of the bottom surface was reported mainly when heavier boxes (10kg) were handled. The preference for upper grip were shown handling light boxes (4.2 kg).

Silva et al, 2013	Two bilateral cutouts (12x4 cm, 5 cm) below the upper edges and two others inserted under in the box surface	44.0x31.0x31.5	EMG, EGM, INC, camera, perceived comfort and grip preference	Upper limbs	The use of box adapted with handles decrease musculoskeletal load in both measurements. The preference was according handing heights.
Nogueira et al, 2016	Two bilateral cutouts (12x4 cm, 5 cm) below the upper edges and two others inserted under in the box surface	44.0x31.0x31.5	EMG, EGM, INC, camera, perceived comfort and grip preference	Upper limbs	The mainly biomechanics effects were identified for inexperienced subjects. Both groups preferred handling tasks using handles.

## Additional Points

*Practitioner Summary*. Manual box handling is considered to cause most of the health-related costs and problems in work environments. Interventions involving box modification by adding handles should have an impact on future boxes' design and improve work conditions.
